# Systematic geriatric assessment for older patients with frailty in the emergency department: a randomised controlled trial

**DOI:** 10.1186/s12877-021-02351-2

**Published:** 2021-07-02

**Authors:** Janne Alakare, Kirsi Kemp, Timo Strandberg, Maaret Castrén, Dimitrije Jakovljević, Jukka Tolonen, Veli-Pekka Harjola

**Affiliations:** 1 Department of Emergency Medicine and Services, University of Helsinki and Helsinki University Hospital, PL 340 |Haartmaninkatu 4, 00029 HUS Helsinki, Finland; 2 University of Helsinki and Helsinki University Hospital, Helsinki, Finland; 3 Centre for Life Course Health Research, University of Oulu, Oulu, Finland; 4 Päijät-Häme Joint Authority for Health and Wellbeing, Services for Older People, Lahti, Finland; 5 Department of Internal Medicine, University of Helsinki and Helsinki University Hospital, Helsinki, Finland

**Keywords:** Frailty, Aged, Aged, 80 and over, Emergency Departments, Geriatric Assessment, Length of Stay

## Abstract

**Background:**

Comprehensive geriatric assessment provided in hospital wards in frail patients admitted to hospital has been shown to reduce mortality and increase the likelihood of living at home later. Systematic geriatric assessment provided in emergency departments (ED) may be effective for reducing days in hospital and unnecessary hospital admissions, but this has not yet been proven in randomised trials.

**Methods:**

We conducted a single-centre, randomised controlled trial with a parallel-group, superiority design in an academic hospital ED.

ED patients aged ≥ 75 years who were frail, or at risk of frailty, as defined by the Clinical Frailty Scale, were included in the trial. Patients were recruited during the period between December 11, 2018 and June 7, 2019, and followed up for 365 days.

For the intervention group, systematic geriatric assessment was added to their standard care in the ED, whereas the control group received standard care only.

The primary outcome was cumulative hospital stay during 365-day follow-up. The secondary outcomes included: admission rate from the index visit, total hospital admissions, ED-readmissions, proportion of patients living at home at 365 days, 365-day mortality, and fall-related ED-visits.

**Results:**

A total of 432 patients, 63 % female, with median age of 85 years, formed the analytic sample of 213 patients in the intervention group and 219 patients in the control group.

Cumulative hospital stay during one-year follow-up as rate per 100 person-years for the intervention and control groups were: 3470 and 3149 days, respectively, with rate ratio of 1.10 (95 % confidence interval, 0.55–2.19, *P* = .78). Admission rates to hospital wards from the index ED visit for the intervention and control groups were: 62 and 70 %, respectively (*P* = .10). No significant differences were observed between the groups for any outcomes.

**Conclusion:**

Systematic geriatric assessment for older adults with frailty in the ED did not reduce hospital stay during one-year follow-up. No statistically significant difference was observed for any secondary outcomes. More coordinated, continuous interventions should be tested for potential benefits in long-term outcomes.

**Trial registration:**

The trial was registered in the ClinicalTrials.gov (registration number and date NCT03751319 23/11/2018).

## Introduction

Frail older adults are a major patient group in emergency departments (ED). Many validated tools that have been developed to recognise and classify frailty are also implemented in ED care [1–4]. For example, the Rockwood Clinical Frailty Scale (CFS) has been validated with good inter-rater reliability for the ED-environment for predicting adverse outcomes. The CFS has been used in emergency care as a quick and feasible tool for frailty status assessment [5–10]. On the other hand, in-depth intervention, comprehensive geriatric assessment (CGA), when provided for admitted hospital patients, has been shown to be effective in reducing mortality and increasing the likelihood of subsequently living at home [11].

It is likely that many frail patients who visit EDs do not receive beneficial interventions which could be effective for long term outcomes. Possible reasons for this include: frailty may not be identified, patients may have multiple ED-visits with different or nonspecific complaints while geriatric syndromes remain disregarded, and organisations may not have services set up for geriatric care. Therefore, EDs may be a practical point for connecting geriatric care needs with a systematic approach.

In previous nonrandomised studies, physician-led CGA provided in acute care settings has been associated with reduced hospital admissions from the ED [12–15]; however, no efficacy for longer term outcomes has been shown. Nurse-led interventions provided after ED visits may be beneficial in preventing functional decline, but results concerning ED-readmissions and hospital admissions are mixed [16–25]. In our opinion, better understanding with the right scope and timing for geriatric interventions for frail older adults visiting EDs is required. We hypothesised that systematic, individualised, multi-dimensional geriatric assessment in frail ED patients would reduce hospital admissions, lengths of stay (LOS) in hospital, and revisits. Furthermore, coordinating good ED care with rehabilitative measures could avoid functional decline, reduce nursing home admissions and ensure better quality of life.

## Methods

### Ethical approval and registration

This trial complied with the ethical rules stated in the Declaration of Helsinki. The study protocol, informed consent forms, and data protection plan were approved by the Ethics Committee II of the Helsinki University Hospital (reference number HUS/1711/2018), and the research permit was issued by the Helsinki University Hospital (reference number HUS/278/2018).

The trial was registered in the ClinicalTrials.gov (registration number and date: NCT03751319, 23/11/2018).

### Trial design

This was a single centre, randomised controlled trial with parallel group, two-arm, superiority design. Recruited patients were randomised to the intervention and the control groups with a 1:1 allocation ratio.

### Setting

The trial was conducted in an academic ED with 60 000 annual adult patient visits. The ED is adjoined both Helsinki University Hospital and Espoo Community Hospital wards located on the same hospital campus. Older frail patients follow general adult patient pathways in the ED. The ED has a geriatric nurse and physical therapist from adjoining community hospital available on demand, to help with mobilisation and rehabilitative measures and to organise home-care. At the time of trial, the ED had no systematic protocol for geriatric assessment, and no specific geriatric care outside the study protocol was in practice in the ED.

### Participants

The eligibility criteria for participants were the following: an ED-visit during the recruitment period; age ≥ 75 years; frail, or at risk of frailty, defined by the CFS level of four to nine; and residency in the hospital service area.

### Enrolment and randomisation

A random sequence for two-group allocation was computer-generated using the online service provided by GraphPad Software. No blocking or any restrictions was applied for sequence generation.

Numbered sealed envelopes with corresponding codes inside (“I” for the intervention group and “C” for the control group) were sealed by an assisting person outside the study group.

Screening for eligibility was done at all hours throughout the study period. The ED secretary assigned an individual code for all patients who met the age and residency criteria, and coded research forms were delivered to patients’ nurses in the ED. Nurses were asked to assess the CFS-grade of the patient, and to obtain background and National Early Warning Score 2 (NEWS2) data if the assessed CFS of the patient was from four to nine. NEWS2 is a structured acute care risk assessment tool based on measured vital signs [26]. Patient enrolment was active during office hours. For eligible patients, written and verbal information about the study was given and consent requested. Consent was obtained from the patient’s relative or caregiver in the ED or through a phone call for those who were not capable of consenting. Eligible consented patients were then enrolled and registered in the study. A health-related quality of life (HRQoL) assessment (the EQ-5D-5 L-questionnaire) was filled in with each patient or with a caregiver, as appropriate. After enrolment, a study physician opened the envelope concordant with the patient’s registration number and filled in the study group in the registration sheet. If a major concern or life-threatening condition was recognised during trial enrolment, it was communicated to the ED team providers.

ED team (ED physician of any speciality and ED nurses) treated both intervention and control group patients according to standard principles for chief complaint and acute condition. No systematic assessment or screening for geriatric conditions or further assessment or care were included in standard care, but if ED team recognised a need for geriatric nurse and physical therapist consultations, it was available.

### Intervention procedures

For the intervention group, systematic geriatric assessment was added to their standard care. Assessment was provided separate from ED team care. While the ED team physician was in charge of the acute care for all enrolled patients, the geriatric assessment was led by second physician assigned for the assessment protocol. Two geriatricians and two emergency physicians with consultation support from a geriatrician were allocated for providing the assessment for the six-month study enrolment period. Assessment was performed in structured form, but with consideration of patient co-operation and abilities for each test.

The study physicians based the evaluation of functioning on activities of daily living and observed the patients’ ability to walk. An orthostatic test was performed when possible. The Abbreviated Mental Test 4 (AMT4) and Six-Item-Screener were used for assessment of cognitive status [27, 28]. The study physicians screened the patients for delirium with the 4 ‘A’s Test (4AT) [29], and for depression with the Patient Health Questionnaire 2 (PHQ-2) [30]. Risk of falls and sarcopenia were evaluated using clinical judgment. Medication reviews were performed with special attention to potential adverse effects, interactions, or undertreatment. Any need for further assessment, testing, or rehabilitation was evaluated. The study physicians interviewed the relatives or caregivers for those patients who could not provide a patient history, with the patient’s consent as appropriate. If the patient could not co-operate, assessments were performed as feasible, and further information was sought from the electronic medical records (EMR).

**Fig. 1:**
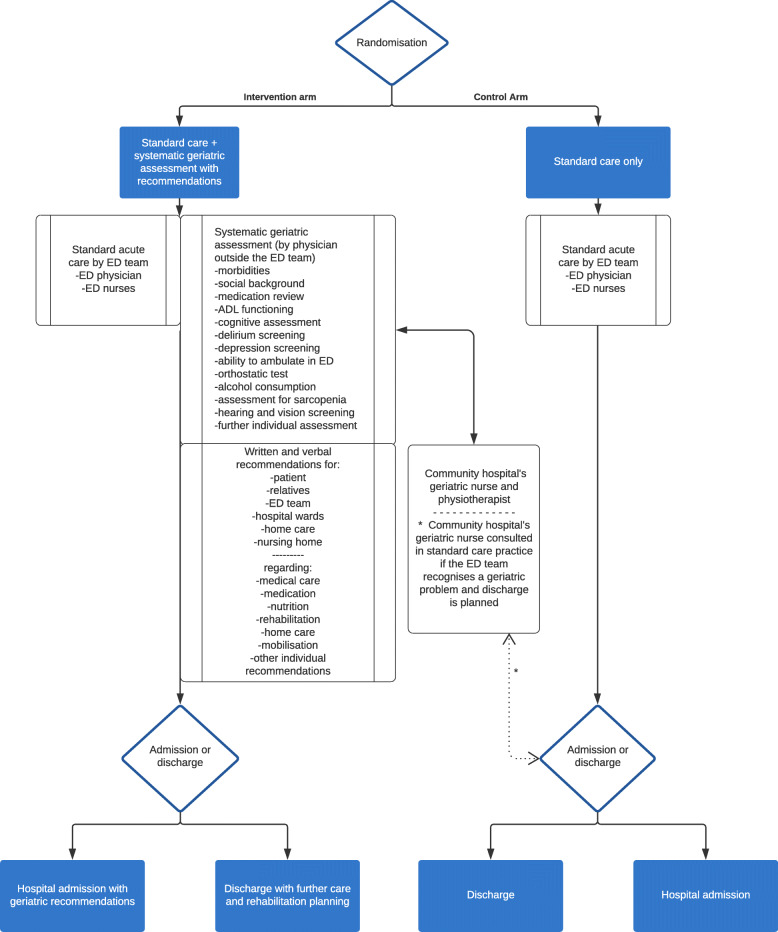
Flowchart of care in the emergency department for trial participants. Abbreviations: ED, emergency department; ADL, Activities of daily living

After completing the assessments, the study physicians provided individual, multi-dimensional recommendations and advice regarding medical care, medications, rehabilitation, nutrition, and evaluation of the need for home-care to the patient, the ED-physician and nurses providing acute care, hospital wards, and home-care. This was done verbally both in the ED and through phone calls, and with structured documentation in the EMR. A Geriatric nurse and physical therapist from community hospital helped to organise support, care, and at-home rehabilitation for discharged patients. Detailed recommendations of mobilisation, nutrition, further assessments, and medical treatment were forwarded to the hospital wards if the patient was admitted. The protocol for trial participants is summarised in Fig. 1.

### Blinding

Due to the active intervention provided in the ED, blinding of patients or personnel was not possible. However, the study physicians did not actively inform the ED personnel providing care for control patients about the patients’ enrolment in the trial. Geriatric assessment and recommendations were documented in the EMR for patient’s future care, so outcome-assessment was not blinded.

### Outcomes

The pre-specified primary outcome measure was the cumulative LOS, as given by the total number of overnight stays in hospital wards (both at the tertiary hospital and the community hospital ward) during 365-day follow-up from the date of the enrolment. All hospital admissions, from the index ED-visit or later, during the follow up period were included. Primary outcome data were retrieved from the tertiary hospital EMR with portal to the community hospital records after follow-up.

The pre-specified secondary outcomes were cumulative number of admissions to hospital wards during the 365-day follow-up time, admission rate from the index visit, readmissions to the ED within 72 h, 30 days, and 365 days after the index visit, proportion of patients alive and home-dwellingat 365 days after the enrolment, and HRQoL-measurement using the EuroQol EQ-5D-5 L instrument [31, 32]. Secondary outcome data were acquired by reviewing the EMR, and by phone interview (with patients, or their caregiver as surrogate) for EQ-5D-5 L with standardised forms designed for phone interview, after the 365-day follow-up time. The EuroQol Crosswalk Index Value Calculator v2 with the Denmark value set was used for EQ-5D-5 L index-value calculations.

The pre-specified outcome measures for assessing the safety and feasibility of the intervention were LOS in the ED on the index-visit, hospital-LOS from the index-visit, 365-day mortality, and number of ED visits related to falls during the follow-up period. Data for these outcomes were obtained by reviewing the EMR. Other prespecified adverse events were not defined.

### Sample size

We based the sample size calculation on an assumed total hospital stay of 25 ± 15 days during the 365-day follow-up time. The assumed hospital stay was an estimation based on previous five-year ED patient data and national reports on inpatient care. A reduction of 10 % in hospital stay was assumed for the intervention group. We calculated a minimum sample size of 392 patients in each group to reach a power of 80 % with 5 % alpha level. A goal was set to recruit a total of 900 patients in order to exceed minimum sample size requirements.

### Statistical methods

For the outcomes depending on 365-day follow-up time, to account for patients who died during the follow-up, total follow-up time contributed by patients in each group were calculated. Rate per 100 person-years and rate ratio estimate with 95 % confidence interval (CI) applying the Byar method were calculated. For the binary outcomes, risk ratios (RR) with 95 % CI were calculated, and the significance of differences were tested with ${\chi }^{2}$tests. For the other outcomes, means with standard deviations (SD) and differences of means with 95 % CI, or medians with inter-quartile range (IQR) and difference of medians were calculated. Significance of mean differences were tested with *t* test and median difference with Mann Whitney U test. A *P* value < 0.05 was considered statistically significant for all outcomes. Intention-to-treat analyses were performed, except for the EQ-5D-5 L-outcome, where only patients with data available were included.

We used the IBM SPSS Statistics for Windows software version 25.0 (Armonk, NY: IBM Corp) for statistical analyses. OpenEpi, Version 3 calculator was used for rate ratio calculations.

## Results

### Participants

**Fig. 2:**
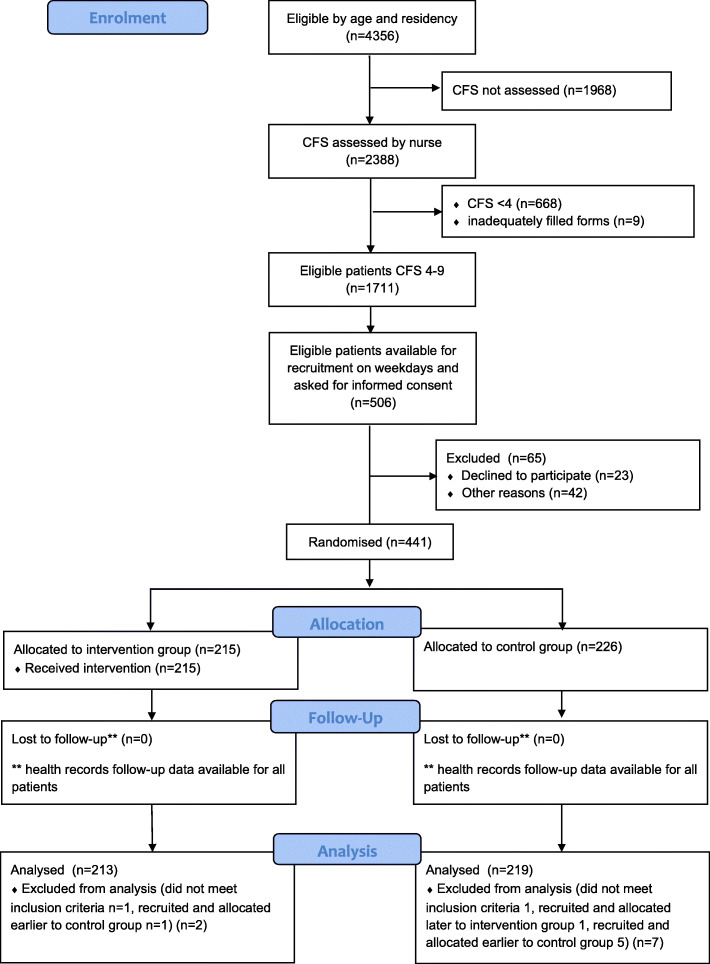
CONSORT 2010 Flow Diagram of enrolment, intervention allocation, follow-up, and data analysis. Abbreviations: CFS, Clinical frailty scale

Patients were enrolled into the trial in a planned six-month period between December 11, 2018 and June 7, 2019. We were unable to enrol the anticipated sample size during the planned time period due to slow recruitment. A total of 4356 patients were identified as eligible for the study based on demographics (Fig. 2). The CFS was assessed for 2388 patients, with 1711 patients assessed as within the eligible CFS-class of four to nine. Of those available during office hours, 506 patients were asked, or their caregiver were reached for, for informed consent, of whom 23 patients (or caregivers) declined, and for 42 patients who were not capable for consenting a caregiver was not available. Randomisation was performed for 441 patients. After enrolment, two patients from the intervention group and seven patients from the control group were excluded (enrolled twice or did not meet inclusion criteria). Ultimately, 213 and 219 patients in the intervention and control groups, respectively, were included in the analysis.

Baseline characteristics for the trial groups are described in Table 1. The median age was 85 (IQR 80–90), the proportion of female patients was 62.7 %, the median CFS was 6 (IQR 5–6), and the proportion of patients living at home (with or without home-care assistance) was 89.7 %.

**Table 1:** Baseline characteristics of the participants

		Intervention group	Control group
		213	219
Age	median (IQR)	85 (80-89.5)	85 (80-90)
	range	75 - 103	75 - 102
Female gender	n (%)	136 (63.8)	135 (61.6)
CFS	median (IQR)	6 (5-6)	5 (5-6)
Home-dwelling^a^	n (%)	194 (91.1)	170 (88.1)
Number of medications^b^	median (IQR)	10 (7-14)	10 (7-14)
Cardiovascular disease	n (%)	123 (57.7)	131 (59.8)
Cognitive disorder	n (%)	82 (38.5)	93 (42.5)
Pulmonary disease	n (%)	43 (20.2)	46 (21.0)
Malignancy	n (%)	28 (13.1)	26 (11.9)
Diabetes	n (%)	54 (25.4)	50 (22.8)
Stroke or TIA in history	n (%)	36 (16.9)	39 (17.8)
ED chief complaint: trauma	n (%)	41 (19.2)	43 (19.6)
ED chief complaint: acute condition nontrauma	n (%)	166 (77.9)	172 (78.5)
ED admission for other or nonspecific cause	n (%)	6 (2.8)	4 (1.8)
NEWS2^c^	median (IQR)	1 (0-3)	1 (0-4)
EQ-5D-5L-index^d^	mean (SD)	0.557 (0.283)	0.545 (0.264)
EQ-5D-5L-VAS^e^	mean (SD)	45.4 (27.1)	41.8 (27.0)

Abbreviations: *IQR* interquartile range, *SD* standard deviation, *NEWS2* National Early Warning Score 2, *ED* emergency department, *VAS* visual analog scale

^a^Home-dwelling status data acquired during the intervention included for the intervention group. Data available for 213 patients in the intervention group and 193 patients in the control group

^b^Number of medications data available for 108 patients in the intervention group and 119 in the control group

^c^NEWS2 data of ED admission available for 180 patients in the intervention group and 193 in the control group

^d^EQ-5D-5L-index -data available for 112 patients in the intervention group and 129 in the control group

^e^EQ-5D-5L-VAS -data available for 118 patients in the intervention group and 139 in the control group

We reviewed the EMR after a 365-day follow-up time for each patient. Survival data were available for all patients. Available data related to hospital stay, mortality, admissions, and home-dwelling status were reviewed for all patients.

### Intervention

In the intervention group, all 213 patients were assessed. For two patients, the assessment was interrupted by admission or discharge, but recommendations were given based on available information.

The study physicians gave and documented at least one recommendation for 202/213 (94.8 %) patients. The most frequent recommendation was about medication (given to 168/213; 78.9 % of the patients), followed by advice on organising home care or rehabilitation-at-home services (65/213; 30.5 %). Clinical findings and recommendations based on assessment are described in Table 2.

**Table 2:** Assessment and recommendations in the intervention group

Intervention group	n	213
**AMT-4**	n (%)	188 (88.3)
	median (IQR)	4 (2-4)
4	n (%)	106/188 (56.4)
3	n (%)	32/188 (17.0)
2	n (%)	24/188 (12.8)
1	n (%)	14/188 (7.4)
0	n (%)	11/188 (5.9)
**Six-Item-Screener**	n (%)	153 (71.8)
	median (IQR)	4 (2-6)
**4AT**	n (%)	186 (87.3)
	median (IQR)	1 (0-3)
**PHQ-2**	n (%)	147 (69.0)
1-2	n (%)	60/147 (40.8)
0	n (%)	87/147 (59.2)
**Orthostatic test**	n (%)	60 (28.2)
orthostatic hypotension	n (%)	20/60 (33.3)
**Alcohol consumption**	n (%)	189 (88.7)
alcohol use (any)	n (%)	35/189 (18.5)
**Sarcopenia or cachexia**	n (%)	209 (98.1)
sarcopenia or cachexia present	n (%)	89/209 (42.6)
**Falls in last 12 months**	n (%)	161 (75.6)
2 or more	n (%)	84/161 (52.2)
1	n (%)	24/161 (14.9)
0	n (%)	53/161 (32.9)
**Recommendations or advice**	n (%)	213 (100.0)
Any	n (%)	202 (94.8)
Medication	n (%)	168 (78.9)
Home support services^a^	n (%)	65 (30.5)
Support or rehabilitation for mobility	n (%)	58 (27.2)
Dietary	n (%)	49 (23)

Abbreviations: *AMT-4* “Abbreviated Mental Test 4”, *4AT* “4 A’2s test for Delirium Screening”, *PHQ-2* “Patient Health Questionnaire-2: Screening Instrument for Depression”

^a^home care, rehabilitation-at-home, further assessment for supportive care

In the control group, two patients received documented geriatric advice related to a major concern noticed during enrolment. These patients were included in the analysis.

### Primary outcome

Patients in the intervention group had cumulative hospital stay of total 6093 days and contributed follow-up time of total 175.6 person-years when mortality during the follow-up was taken into account. Patients in the control group had cumulative hospital stay of total 5734 days and contributed follow-up time of total 182.1 person-years. Hospital stay rate during the 365-day follow up per 100 person-years for the intervention and control group were: 3470 and 3149 days, respectively, with rate ratio of 1.10 (95 % CI 0.55–2.19), without statistically significant difference (*P* = .78). (Table 3).

**Table 3:** Clinical outcomes of the trial

Outcome		Intervention *n* = 213	Control *n* = 219	ratio or difference [95 % CI]	*p* value
**Primary outcome**					
Cumulative hospital stay(days) during 365d follow-up	rate per 100 person-years	3470	3149	rate ratio 1.10 [0.55;2.19]	0.783
**Secondary outcomes**					
Admission from ED index visit	n (%)	133 (62.4)	153 (69.9)	risk ratio 0.89 [0.78;1.02]	0.103
Number of hospital admissions30d	mean (SD)	0.79 (0.60)	0.91 (0.64)	mean difference − 0.12 [-0.23;0.00]	0.475
Hospital admissions 365d	rate per 100 person-years	230	246	rate ratio 0.94 [0.82;1.07]	0.345
Number of ED readmissions 72 h	mean (SD)	0.04 (0.19)	0.05 (0.21)	mean difference − 0.01 [-0.05;0.03]	0.400
Number of ED readmissions 30d	mean (SD)	0.29 (0.58)	0.29 (0.54)	mean difference 0.00 [-0.11;0.11]	0.817
ED readmissions 365d	rate per 100 person-years	268	238	rate ratio 1.12 [0.99;1.28]	0.081
Alive and living-at-home 365d	n (%)	116 (54.5)	111 (50.7)	RR 1.07 [0.90;1.29]	0.432
EQ-5D-5 L-index 365d^a^	mean (SD)	0.53 (0.28)	0.53 (0.27)	mean difference 0.0 [-0.06;0.06]	0.995
EQ-VAS 365d^b^	mean (SD)	55.28 (23.83)	53.23 (22.78)	mean difference 2.04 [-3.26;7.35]	0.449
**Other outcomes**					
ED LOS hh:mm	median (IQR)	7:23 (5:22 − 15:23)	9:25 (6:00–18:40)	median difference − 2:02	0.052
Hospital LOS from the index-visit if admitted (days)^c^	mean (SD)	15.91 (24.30)	12.30 (20.38)	mean difference 3.61 [-1.67;8.83]	0.069
Death 365d	n (%)	53 (24.9)	59 (26.9)	risk ratio 0.92 [0.67;1.27]	0.626
Death 30d	n (%)	16 (7.5)	15 (6.8)	risk ratio 1.10 [0.56;2.16]	0.790
ED visits for falls 365d^d^	rate per 100 person-years	59	47	rate ratio 1.27 [0.95;1.69]	0.104

Abbreviations: *CI* Confidence Interval, *ED* Emergency Department, *SD* Standard Deviation, *LOS* Length of Stay, *IQR* interquartile range

^a^data available for 150 patients in the intervention group, 150 patients in the control group

^b^data available for 149 patients in the intervention group, and 150 patients in the control group

^c^Only patients admitted from the index ED visit included. Only the first hospital stay period counted for LOS.

^d^patients who visited the ED after the index ED visit for fall-related complaint

### Secondary outcomes

The proportion of hospital admissions from index visit was 7.5 % point lower in the intervention group (133/213; 62.4 %) than in the control group (153/219; 69.9 %), but without statistical significance, RR 0.89 (95 % CI, 0.78–1.02; *P* = .10). During the 30-day follow-up, means of number of hospital admissions for the intervention and the control group were similar: 0.79 (SD 0.60) and 0.91 (SD 0.64), respectively, with mean difference of -0.12 (95 % CI, -0.23–0.00; *P* = .48). During the 365-day follow-up hospital admission rates did not differ significantly: 230 admissions per 100 person-years in the intervention group and 246 in the control group, with rate ratio of 0.94 (95 % CI, 0.82–1.07; *P* = .35).

Readmissions to the ED for the intervention and control group did not significantly differ. In the 72-hour and 30-day follow-up number of ED-readmissions had means of 0.04 (SD 0.19) vs. 0.05 (SD 0.21), difference − 0.01 (95 % CI, -0.05–0.03, *P* = .40); and 0.29 (SD 0.58) vs. 0.29 (SD 0.54), difference 0.00 (95 % CI, -0.11–0.11, *P* = .82), respectively. Rates of ED readmission in 365-day follow-up per 100 person-years were: 268 and 238 for the intervention and control group, respectively, with rate ratio of 1.12 (95 % CI, 0.99–1.28), *P* = .08.

The proportions of patients alive and living at home at the end of 365-day follow-up were: 116/213 (54.5 %) in the intervention group, and 111/219(50.7 %) in the control group; RR 1.07 (95 % CI, 0.90–1.29; *P* = .43).

A total of 300 EQ-5D-5 L-index follow-up assessments were completed, of which 186 answered by patients and 114 by caregivers. The EQ-5D-5 L-VAS question was answered for 299 patients. The EQ-5D-5 L-index for the intervention and the control group after 365-day follow-up were mean 0.53 (SD 0.28) and 0.53 (SD 0.27), respectively (difference 0.00, 95 % CI, -0.06–0.06; *P* > .99), and the EQ-5D-5 L-VAS values were mean 55.3 (SD 23.8) and 53.2 (SD 0.22.8), respectively (difference 2.0; 95 % CI, -3.3–7.4; *P* = .45).

The ED-LOS between the groups did not significantly differ: median 7:23 h:minutes (IQR 5:22 − 15:23) and 9:25 h:minutes (IQR 6:00–18:40), for the intervention and the control groups, respectively (*P* = .05). Hospital-LOS for patients admitted from the index-ED-visit were: mean 15.91 (SD 24.30) days for the intervention group and 12.30 (20.38) for the control group, mean difference 3.61 (95 % CI, -1.67–8.83), *P* = .07. The 30-day and 365-day mortalities were similar between the groups: 30-day mortality was 16/213 (7.5 %) for the intervention group, and 15/219 (6.8) for the control group, RR 1.10 (95 % CI, 0.56–2.16; *P* = .79), and 365-day mortality was 53/213 (24.9 %) for the intervention group, and 59/219 (26.9 %) for the control group, RR 0.92 (95 % CI, 0.67–1.27; *P* = .63). Rates per 100 person-years for ED visit related to falls during 365-day follow-up were: 59 for the intervention group and 47 for the control group, with rate ratio of 1.27 (95 % CI, 0.95–1.69; *P* = .10).

## Discussion

In this study, the anticipated reduction in hospital stay was not achieved with systematic geriatric assessment provided in the ED. Hospital admission rate from the index visit was lower in the intervention group without an increased number of ED-revisits, but without statistical significance. There was no significant difference in the ED-LOS, hospital-LOS after the index visit, hospital admission rates, 365-day mortality, ED-revisits for falls, home-dwelling status, or HRQoL at the end of the follow-up, between the intervention and the control groups.

There are many possible explanations for the null results. First, study patients were heterogeneous with some of them possibly having no chance of benefiting from the intervention due to, for example, terminal illness. However, most patients were home-dwelling at baseline with multi-morbidity and impairment in daily functions, so it can be argued that the patient population in this trial should benefit from the intervention. As yet, no adapted, more specific criteria exist for targeting CGA for patients in the ED. It is challenging to find efficient interventions for this heterogeneous patient population in the complex environment of emergency care.

Second, the deviance in the cumulative hospital stay was large, and only a small proportion of patients contributed the major proportion of the hospital stay (42 % of total hospital-stay days of the study population were occupied by patients in the highest decile in cumulated hospital stay). It is possible that home-care-based implementation of care and rehabilitation are insufficient for patients who are vulnerable to prolonged hospital stay.

Third, patients in this study were cared for in many different organisations in the tertiary hospital, community hospital, home care, outpatient clinics and nursing homes. Delivering recommendations to patients and other institutions in written and verbal form was emphasised in the intervention, but it is not known how thoroughly these recommendations were implemented.

Fourth, standard treatment in EDs probably varies according to how well geriatric syndromes and frailty-related issues are taken into account, and how geriatric care is organised. In the ED of this study, no systematic assessment protocol for geriatric problems existed in standard care, but awareness of good geriatric care may have been prevalent, and many services from community hospital including geriatric nurse were available for control group patients if need for such service was recognised. Therefore, some dilution of the efficacy may have occurred.

Identifying frail patients in hospital wards remain crucial for early initialisation of hospital-based-CGA, which has been shown to be efficient [11]. Therefore, assessment for frailty status in the ED may be a route towards more comprehensive care, but, compared to intervention in this trial, more intensive and continuous ward-based interventions may be needed for admitted patients. Efficient interventions remain unknown for those frail patients who are discharged from the ED. Similar results to this trial were reported in a randomised trial in which CGA was provided after discharge from acute medical units [33].

In previous, nonrandomised studies, the admission rate of older patients from ED was reduced by ED-related CGA by 2.6–19.7 % points [34]. In our study, patients in the intervention group were less often admitted to hospital, with no difference in ED-revisit rate. The reduction of admission rate did not reach statistical significance here, but, in the light of previous studies, the efficacy of intervention for this outcome is likely to be real. For admitted patients, the hospital-LOS was longer. The intervention might have altered patient selection for admission, increasing the hospital LOS. It is also possible that recommended further assessments and interventions contributed for longer LOS. Assuming that unnecessary hospital admissions can be safely avoided with geriatric interventions in the ED, it remains to be demonstrated whether this effect prevails in a longer period when the same patients have multiple ED-visits. Considering the risk of hospitalisation-acquired disability, promoting discharge for patients who do not have an absolute need for hospital care is likely to be beneficial for the patients [35].

Overall, the findings of our study concur with late reviews assessing ED-based interventions for older adults [25, 36]. Individualised, more effective geriatric interventions for frail patients in the EDs, and detailed identification for patients who benefit from such interventions, remain to be studied.

### Strengths and limitations

This study has many strengths. The randomised, controlled trial protocol enables a robust assessment of the efficacy of the intervention. For the primary outcome, and most of the secondary and other outcomes, reliable outcome data were available from the EMR. The trial protocol was implemented in a real-life environment with typical frail patients in the ED. Furthermore, our mid-sized ED, with its nonselected adult patient population, can be considered a typical acute care setting.

Besides the nonblinded protocol, a major limitation in this trial was that enrolment did not fulfil the expected sample size. An important reason for this was that the enrolment process was more laborious than anticipated. Patients had acute care underway so availability for trial enrolment and intervention often had to wait, slowing recruitment. However, this reflects the real challenges when providing geriatric assessment amidst ongoing acute care, and is a real-life feasibility issue in the ED environment. There was not trend of fewer hospital-stay-days in 365-day follow-up for the intervention group, so the reduced sample size is probably not the reason for the null result for the primary outcome.

The CFS was assessed only for 55 % of patients who met the demographic criteria for the study, probably because nursing staff were occupied due to crowding. Study enrolment was active only during office-hours when a study physician was available. Thus, patients enrolled for the study may not be fully representative of frail older patients who visit the ED in late hours or during weekends.

Hospital-stay data was reviewed for all patients, but it is possible that some patients may have been admitted outside our hospital network. However, in Finland, private in-patient hospital beds outside public health care are negligible in number, and we found no documentation of such admissions in the patient records. Therefore, the hospital admission data can be considered conclusive.

Standard care by ED teams was focused on acute conditions, but it is possible that some “leakage” of intervention affected the control group indirectly when personnel was aware of the ongoing trial and may have put more emphasis on geriatric issues in patient care.

## Conclusions

Systematic geriatric assessment and recommendations for older adults with frailty in the ED did not reduce cumulative hospital stay during one-year follow-up. No significant differences were found between the intervention and the control groups in the secondary outcomes of hospital admissions, revisits to the ED, living-at-home status, quality-of-life, or other outcomes.

## Data Availability

The datasets generated and analysed during the trial are not publicly available due national juridical restrictions protecting pseudonymised research data. Based on national jurisdiction ethical board or organisation’s study permission does not allow sharing pseudonymised trial data. Further description or analysis of data are however available from the authors upon reasonable request.

## Abbreviations

AMT4: Abbreviated mental test 4
AT4: 4 'A's test
CFS: Clinical Frailty Scale
CGA: Comprehensive geriatric assessment
CI: Confidence interval
ED: Emergency department
EMR: Electronic medical records
HRQoL: Health-related quality of life
IQR: Inter-quartile range
LOS: Length of stay
NEWS2: National early warning score 2
PHQ-2: Patient health questionnaire 2
RR: Risk ratio
SD: Standard deviation
